# Prognostic value of total tumor volume in patients with nasopharyngeal carcinoma treated with intensity-modulated radiotherapy

**DOI:** 10.1186/s12885-017-3480-5

**Published:** 2017-07-28

**Authors:** Shao-Bo Liang, Jian-Jian Teng, Xue-Feng Hu, Xing-Li Yang, Min Luo, Xiao-Na Fang, Dong-Sheng Liu, Yong Chen, Li-Wu Fu

**Affiliations:** 1 State Key Laboratory of Oncology in South China, Sun Yat-sen University Cancer Center, Collaborative Innovation Center for Cancer Medicine, Guangzhou, 510060 China; 2 Department of Radiation Oncology, Cancer Center, First People’s Hospital of Foshan Affiliated to Sun Yat-sen University, Foshan, China; 3 Department of Medical Statistics, First People’s Hospital of Foshan Affiliated to Sun Yat-sen University, Foshan, China; 4 Department of Radiation Oncology, The First Affiliated Hospital of Sun Yat-Sen University, Guangzhou, China

**Keywords:** Nasopharyngeal carcinoma, Intensity-modulated radiotherapy, Tumor volume, Treatment failure, Staging system

## Abstract

**Background:**

Few studies have evaluated the prognostic value of total tumor volume (TTV), which reflects both the primary tumor volume and nodal tumor volume, in NPC. Furthermore, the relationship between TTV and survival remains unknown. The purpose of this study was to evaluate the prognostic value of TTV in patients with NPC treated with intensity-modulated radiation therapy (IMRT).

**Methods:**

TTV was retrospectively assessed in 455 patients with newly diagnosed, non-metastatic NPC. All patients were treated using IMRT; 91.1% (288/316) of patients with stage III-IVb also received cisplatin-based chemotherapy. Receiver operating characteristic (ROC) curves were used to identify the optimal TTV cut-off point and examine the prognostic value of combined TTV with current clinical stage.

**Results:**

Mean TTV was 11.1 cm^3^ (range, 0.3–27.9 cm^3^) in stage I, 22.5 cm^3^ (1.3–92.4 cm^3^) in stage II, 40.6 cm^3^ in stage III (3.2–129.2 cm^3^), and 77.5 cm^3^ in stage IVa-b (7.1–284.1 cm^3^). For all patients, the 4-year estimated FFS, OS, DMFS, and LRRFS rates for patients with a TTV ≤ 28 vs. > 28 cm^3^ were 93 vs. 71.4% (*P* < 0.001), 95.1 vs. 75.4% (*P* < 0.001), 94.5 vs. 79.4% (*P* < 0.001), and 96.2 vs. 88% (*P* = 0.001). TTV was an independent prognostic factor for FFS, OS, DMFS and LRRFS in all patients. In stage III-IVb, 4-year estimated FFS, OS, DMFS, and LRRFS for a TTV ≤28 vs. >28 cm^3^ were 88.9 vs. 70.5% (*P* = 0.001), 96.2 vs. 72.7% (*P* < 0.001), 91.2 vs. 78.3% (*P* = 0.008), and 93.8 vs. 87.6% (*P* = 0.063). TTV was an independent prognostic factor for FFS, OS and DMFS in stage III-IVb. Receiver operating characteristic (ROC) curve analysis curves revealed adding TTV to clinical stage had superior prognostic value for treatment failure compared to clinical stage alone (*P* = 0.016).

**Conclusions:**

TTV is an important prognosticator for treatment outcome and significantly improves the prognostic value of the current staging system for patients with NPC treated with IMRT.

## Background

Based on GLOBOCAN estimates, there were an estimated 86,700 new cases of nasopharyngeal carcinoma (NPC) and 50,800 associated deaths worldwide in 2012 [1]. The geographic distribution of NPC is extremely unbalanced, with a very low incidence in most regions of the world and high incidence in China and other countries in Southeastern Asia [1, 2]. Radical radiotherapy (RT) is the first treatment choice for non-metastatic NPC and the addition of concomitant chemotherapy to RT provides a significant survival benefit in locoregionally advanced NPC [3].

The overall survival (OS) of patients with NPC has significantly improved in recent years due to widespread application of magnetic resonance imaging (MRI), improvements in RT techniques and the combination of RT with concomitant chemotherapy [4–6]. The 5-year estimated OS rate is currently about 80%, while treatment failure remains the predominant cause of death; 5-year local control ranges from 86 to 95%, 5-year nodal control from 92 to 97% and 5-year distant control from 82 to 85% [7–12].

Accurate prognostication is critical when deciding treatment strategies. Tumor volume is a significant independent prognostic factor in most cancers, including oral carcinoma, B-cell lymphoma and rhabdomyosarcoma [13–15]. Several studies have confirmed the primary tumor volume (PTV) has high prognostic value for survival in NPC [16, 17]. However, few studies have evaluated the prognostic value of the total tumor volume (TTV), which incorporates both the PTV and nodal tumor volume (NTV), in NPC and the relationship between the TTV and survival remains unknown.

Therefore, we initiated a retrospective, large cohort study to evaluate the prognostic value of TTV in patients with NPC treated with intensity-modulated radiation therapy (IMRT), and assessed whether the prognostic validity of the current staging system for NPC could be improved by incorporating assessment of the TTV. We hope this information may help to further clarify the biological characteristics of NPC and guide the design of individual treatment strategies.

## Methods

### Patient characteristics

The Institutional Review Board of First People’s Hospital of Foshan Affiliated to Sun Yat-sen University approved this retrospective study; as this was an analysis of routine clinical data, an exemption from requiring written informed consent was granted. The authenticity of this article has been validated by uploading the key raw data onto the Research Data Deposit public platform (www.researchdata.org.cn), with the approval RDD number as RDDA2017000217. A total of 455 patients with newly diagnosed, non-metastatic NPC treated by IMRT at First People’s Hospital of Foshan Affiliated to Sun Yat-sen University from April 2010 to March 2014 were enrolled in this study [18]. The patients included 347 (76.3%) males and 108 (23.7%) females. The median age was 45 years (17–80 years). All cases had the non-keratinizing pathological type.

Pretreatment examinations included a medical history, physical examination, hematology and biochemistry profiles, electrocardiogram, chest X-ray, abdominal ultrasound, nasal endoscopy and biopsy, pathological examination of the primary tumor, bone scan, and MRI of the nasopharynx and neck. All patients were restaged using the 7th edition of the American Joint Commission on Cancer staging system (AJCC) [19]. The stage/category distribution for the entire cohort was as follows: 127/455 (27.9%) in T1, 59 (13.0%) in T2, 157 (34.5%) in T3 and 112 (24.6%) in T4; 58 (12.7%) in N0, 255 (56.0%) in N1, 119 (26.2%) in N2 and 23 (5.1%) in N3; 29 (6.4%) in stage I, 110 (24.2%) in stage II, 184 (40.4%) in stage III and 132 (29.0%) in stage IVa-b.

### Tumor volume measurement

The patients were immobilized in a supine position using a thermoplastic mask extending from the head to shoulders. CT simulation (Brilliance Big Bore, Phillips, Amsterdam, Netherlands) was performed at a slice thickness of 3 mm from the head to 2 cm below the sternoclavicular joint. The control CT and contrast-enhanced CT images were transferred to the inverse IMRT planning system (Version 8.6, Eclipse, Varian, CA, USA). Tumor volumes were delineated by a radiation oncologist, and verified by another radiation oncologist who specializes in NPC treatment.

**Fig. 1:**
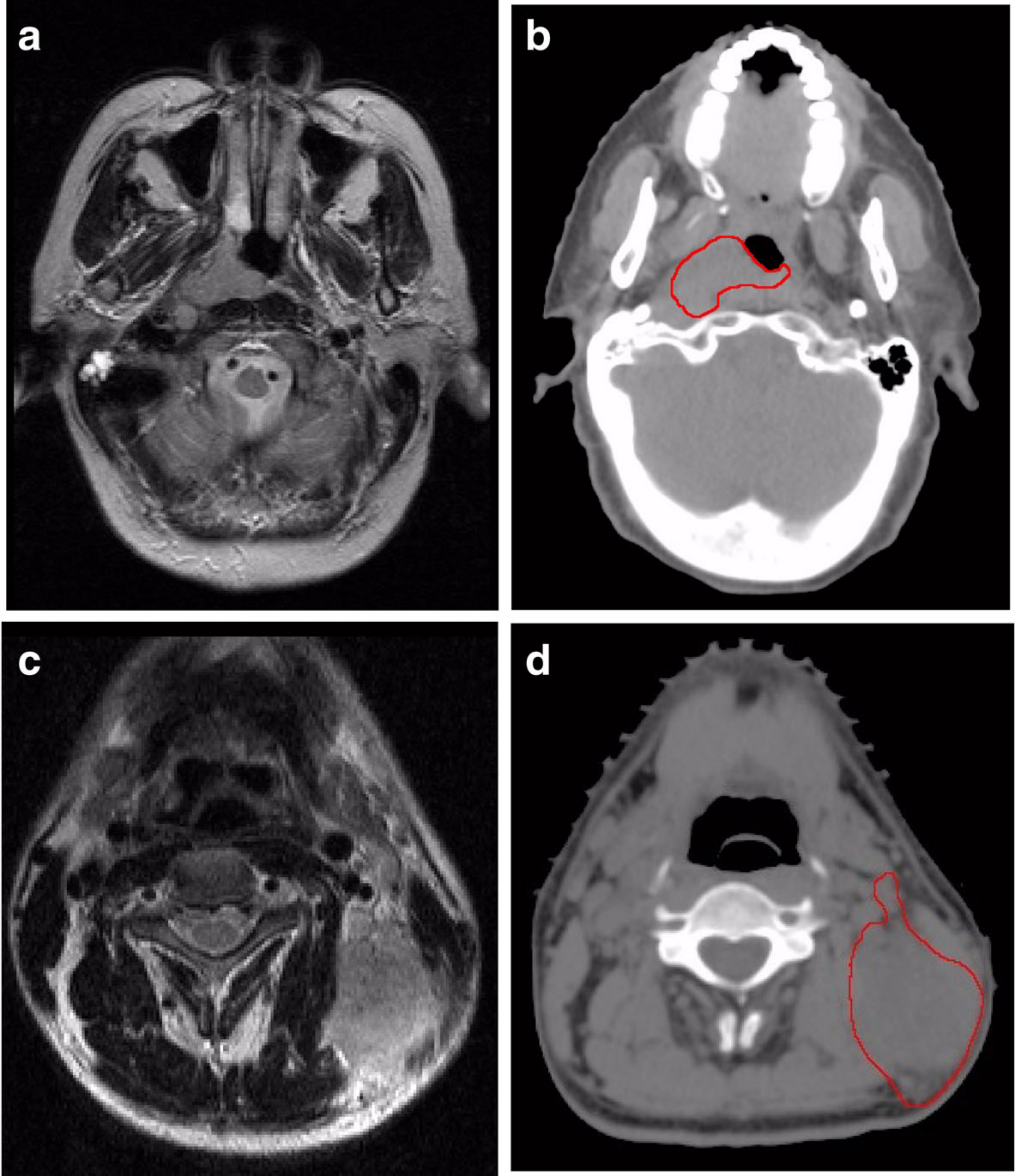
The delineation of PTV and NTV. **a** Axial T2-weighted image illustrating that the primary tumor and retropharyngeal lymph node involvement are located close together, making it difficult to distinguish between them. **b** Control CT image showing the PTV, including the primary tumor and retropharyngeal lymph node involvement, which was delineated according to the pretreatment MRI shown in Fig. 1a. **c** Axial T2-weighted image of neck lymph nodes with extracapsular spread, which was diagnosed on the basis of an irregular border and invasion into the adjacent fat and muscle. **d** Control CT image showing the NTV including metastatic cervical lymph nodes and nodal extracapsular spread, which was delineated according to the pretreatment MRI shown in Fig. 1c

The PTV and NTV were both delineated on the planning system according to the pretreatment MRI. The PTV included the primary tumor and retropharyngeal lymph node (RLN) involvement as these anatomical sites are so close that it remains difficult to distinguish between them (Fig. 1a-b) [16, 20, 21]. The NTV included metastatic cervical lymph nodes (CLN) and nodal extracapsular spread (Fig. 1c-d). The metastatic lymph nodes were diagnosed based on the criteria recommended by Van et al. and Mao et al. [22, 23]. The diagnostic criteria for nodal extracapsular spread included blurred margins or irregular capsular enhancement of lymph nodes, or tumor invasion into adjacent fat and muscle (Fig. 1c). The PTV and NTV were automatically calculated using a shape-based interpretation algorithm, which is obtained by tri-linear interpolation of a stack of two-dimensional distance transforms of transaxial shapes. The TTV was obtained by summing the PTV and NTV.

### Treatment

All patients were treated using IMRT. Target volumes were delineated according to the RTOG IMRT protocols [18]. The planning target volume of the clinical target volume (CTV)70 received 70 Gy in 33 fractions at 2.12 Gy per fraction. Small-volume lymph nodes received 63 Gy in 33 fractions at 1.9 Gy per fraction. The planning target volume of the CTV59.4 received 59.4 Gy in 33 fractions at 1.8 Gy per fraction. The planning target volume of the CTV50.4 received 50.4 Gy in 28 fractions at 1.8 Gy per fraction. RT was delivered over one fraction daily, 5 days per week.

Based on the treatment guidelines for NPC at our hospital, concurrent chemotherapy was recommended to patients with stage T1–2N1M0 and concurrent chemotherapy +/− induction chemotherapy or adjuvant chemotherapy to patients with stage III-IVb NPC. In total, 82 (82/107, 76.6%) patients with clinical stage T1–2N1M0 and 288 (288/316, 91.1%) patients with stage III-IVb received chemotherapy. Induction chemotherapy or adjuvant chemotherapy was consisted of cisplatin (80 mg/m^2^) and fluorouracil (1000 mg/m^2^ daily for 4 days); docetaxel (75 mg/m^2^) and cisplatin (75 mg/m^2^); or a triplet of docetaxel (60 mg/m^2^), cisplatin (60 mg/m^2^) and fluorouracil (800 mg/m^2^ daily for 4 days) every 3 weeks for 2–3 cycles. Concurrent chemotherapy was consisted of cisplatin given every 3 weeks (100 mg/m^2^) or weekly (40 mg/m^2^) during RT. In the event of documented relapse, salvage treatments including RT, surgery or chemotherapy were provided when appropriate.

### Follow up and statistical analysis

After RT, all patients were assessed every 3 months during the first 2 years, and every 6 months thereafter until death. The median follow-up for the entire cohort was 53 months (range, 2 to 83 months). Overall, 439 patients (439/455, 96.5%) received regular follow-up until death or latest scheduled assessment. Failure free survival (FFS) was calculated from assignment to the first failure at any site, OS to death from any cause, distant metastasis-free survival (DMFS) to first remote failure, and loco-regional relapse free survival (LRRFS) to first locoregional failure.

Stata Statistical Package (STATA 11; StataCorp LP, College Station, TX, USA) was used for all analysis. The Kruskal-Wallis test was used to examine the differences in TTV between stages. Actuarial rates were calculated using the Kaplan-Meier method and compared using the log-rank test. Multivariate analyses with the Cox proportional hazards model were used to test for significant independent prognostic factors using a backward elimination strategy. All patients were randomly allocated to a training set (*n* = 152) or test set (*n* = 303). Receiver operating characteristic (ROC) curve analysis was used to evaluate different cut-off points for TTV in the training set. Then, the test set and all patients were stratified according to the optimal cut-off point. The area under the ROC curve was used to assess the prognostic validity of the TTV. The criterion for statistical significance was set at α = 0.05; *P*-values were based on two-sided tests.

## Results

### Distribution of tumor volume by category/stage

**Fig. 2:**
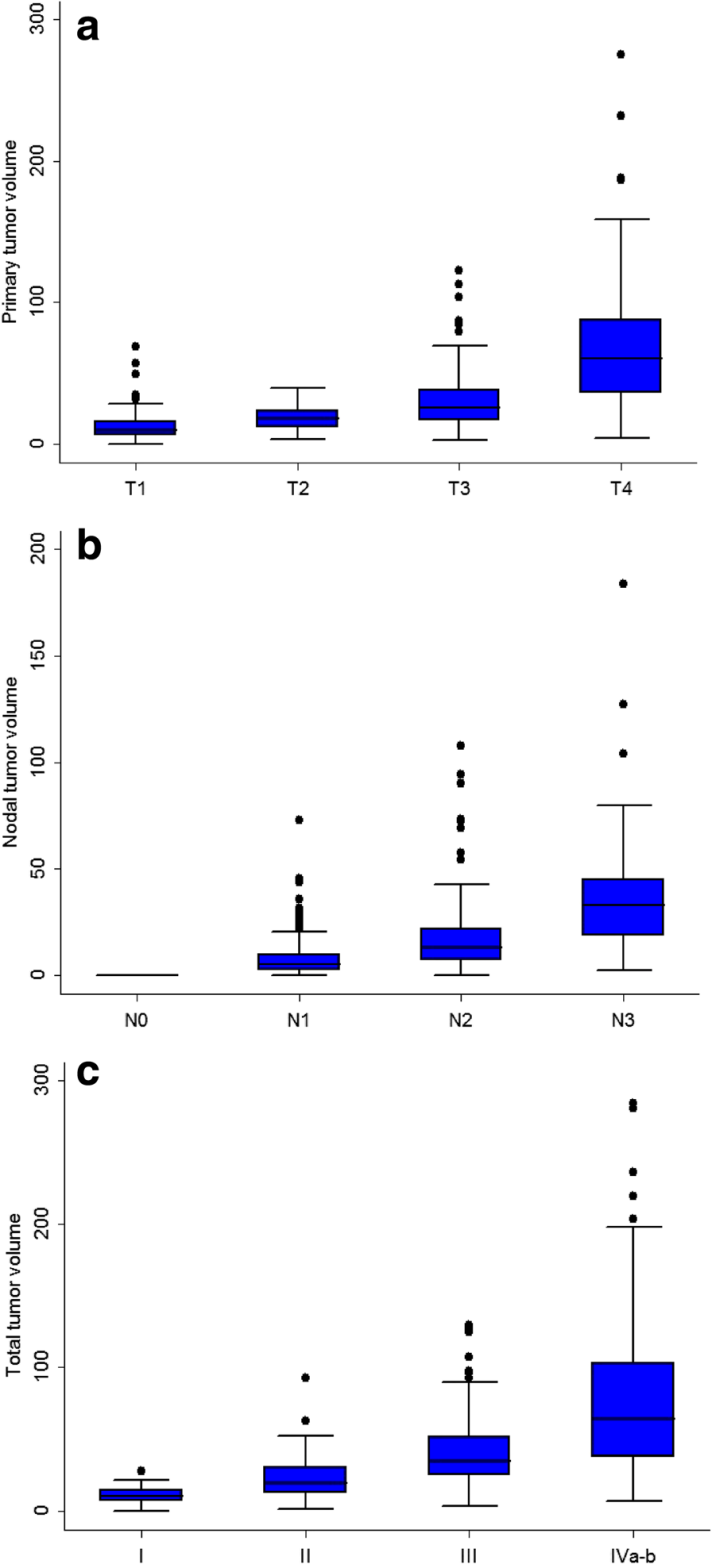
Distribution of tumor volume by stage for all 455 patients. **a** Distribution of primary tumor volume by T category. **b** Distribution of nodal tumor volume by N category. **c** Distribution of total tumor volume by clinical stage

The distribution of PTV stratified by T category is presented in Fig. 2a. The mean PTV was 12.7 cm^3^ (range, 0.3–69.2 cm^3^) in T1, 18.9 cm^3^ (3.2–40 cm^3^) in T2, 30.7 cm^3^ in T3 (2.4–122.5 cm^3^), and 68.7 cm^3^ in T4 (4.1–275.3 cm^3^). The distribution of NTV by N category is presented in Fig. 2b. The mean NTV was 8.0 cm^3^ (0–72.9 cm^3^) in N1, 18.4 cm^3^ in N2 (0.3–107.5 cm^3^), and 44.7 cm^3^ in N3 (2.4–184.0 cm^3^). The distribution of TTV by clinical stage is presented in Fig. 2c. The mean TTV was 11.1 cm^3^ (range, 0.3–27.9 cm^3^) in stage I, 22.5 cm^3^ (1.3–92.4 cm^3^) in stage II, 40.6 cm^3^ in stage III (3.2–129.2 cm^3^), and 77.5 cm^3^ in stage IVa-b (7.1–284.1 cm^3^).

### Identification and verification of TTV cut-off point

With respect to FFS, the optimal cut-off point for the TTV was 28 cm^3^ in the training set (sensitivity 95.7%, specificity 50.4%; area under the ROC curve [AUC] = 0.73, *P* = 0.001). Therefore, we selected 28 cm^3^ as a uniform cut-off point (≤ 28 vs. > 28 cm^3^) in order to classify the test set and all patients into high and low TTV groups for survival analysis.

In the test set (*n* = 303), the 4-year estimated FFS, OS, DMFS, and LRRFS rates for patients with a TTV ≤ 28 vs. > 28 cm^3^ were 90.9 vs. 69.8% (*P* < 0.001), 95.1 vs. 75.1% (*P* < 0.001), 93.4 vs. 77.8% (*P* < 0.001), and 95.8 vs. 88.1% (*P* = 0.005), respectively.

### Prognostic significance of TTV in all patients

**Fig. 3:**
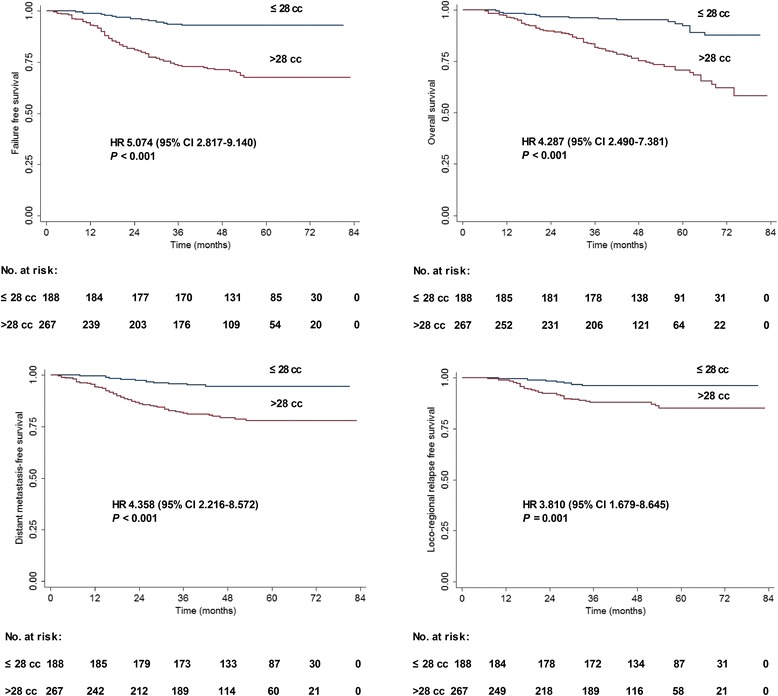
Survival rates for all 455 patients stratified by TTV. **a** Failure-free survival. **b** Overall survival. **c** Distant metastasis-free survival. **d** Loco-regional relapse-free survival

The clinical characteristics of the 455 patients with NPC stratified by TTV ≤ 28 cm^3^ and >28 cm^3^ are shown in Table 1. In all patients (*n* = 455), the 4-year estimated FFS, OS, DMFS, and LRRFS rates for patients with a TTV ≤ 28 vs. > 28 cm^3^ were 93 vs. 71.4% (*P* < 0.001), 95.1 vs. 75.4% (*P* < 0.001), 94.5 vs. 79.4% (*P* < 0.001), and 96.2 vs. 88% (*P* = 0.001), respectively (Fig. 3).

**Table 1:** Clinical characteristics of 455 patients with TTV ≤ 28 and TTV > 28 cm^3^

Characteristics	TTV ≤ 28 cm^3^ (*N* = 188)	TTV > 28 cm^3^ (*N* = 267)	*P* Value^†^
Sex (%)			<0.001
Male	127	220	
Female	61	47	
Age (years)			0.908
≤ 45 years	82	115	
> 45 years	106	152	
T-category^a^ (%)			<0.001
T1	93	34	
T2	31	28	
T3	52	105	
T4	12	100	
N-category^a^ (%)			<0.001
N0	43	15	
N1	116	139	
N2	26	93	
N3	3	20	
Stage-group^a^ (%)			<0.001
I	29	0	
II	78	32	
III	66	118	
IVa–b	15	117	
Chemotherapy			
Yes	139	245	<0.001
No	49	22	
Additional boost			
Yes	28	22	0.025
No	160	245	

*TTV* total tumor volume; † *P* values were calculated by the Chi-square test; ^a^According to the 7th edition of the American Joint Commission on Cancer staging system

The following parameters were included in the Cox proportional hazards model: age (≤ 45 vs. > 45 years), sex (male vs. female), T category (T1–2 vs. T3–4), N category (N0–1 vs. N2–3), chemotherapy (yes vs. no), additional boost (yes vs. no) and TTV (≤ 28 vs. > 28 cm^3^). TTV was an independent prognostic factor for FFS, OS, DMFS and LRRFS in all patients (all *P* < 0.05; Table 2).

**Table 2:** Multivariate analyses of prognostic factors in all 455 patients

Endpoint	Variable	HR	95% CI	*P*-value
FFS	TTV	4.523	2.482–8.241	<0.001
	N stage^a^	1.567	1.027–2.391	0.037
OS	Sex	1.661	0.915–3.013	0.095
	Chemotherapy	1.882	0.986–3.592	0.055
	T stage^a^	1.763	1.054–2.951	0.031
	N stage^a^	1.920	1.254–2.939	0.003
	TTV	3.231	1.776–5.878	<0.001
DMFS	N stage^a^	1.764	1.064–2.925	0.028
	TTV	3.749	1.877–7.489	<0.001
LRRFS	TTV	3.810	1.679–8.645	0.001

^a^According to the 7th edition of the American Joint Commission on Cancer staging system; *HR* hazard ratio, *CI* confidence interval, *FFS* failure free survival, *OS* overall survival, *DMFS* distant metastasis-free survival, *LRRFS* loco-regional relapse free survival, *TTV* total tumor volume

### Prognostic significance of TTV in stage III-IVb NPC

**Fig. 4:**
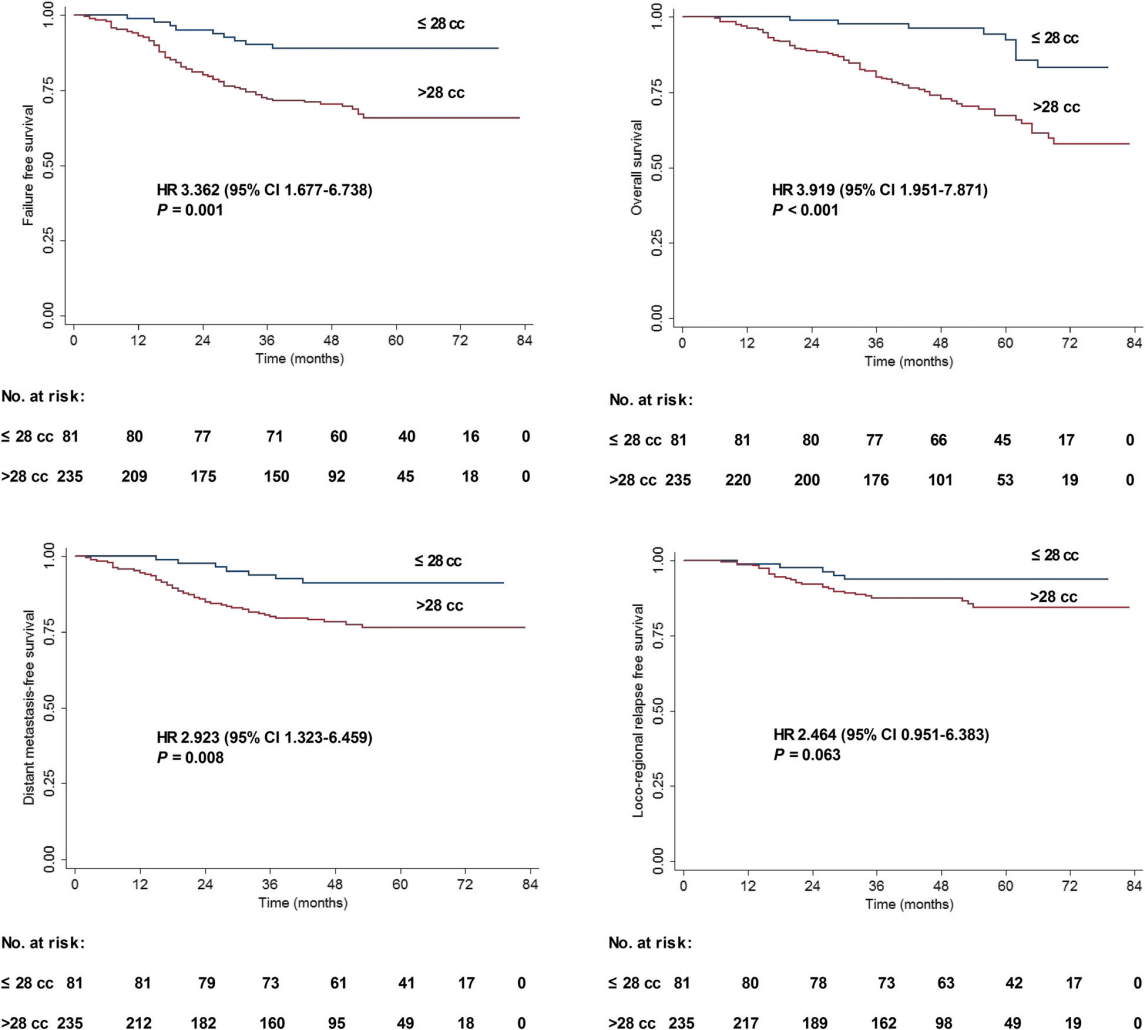
Survival rates for the 316 patients with stage III-IVb stratified by TTV. **a** Failure-free survival. **b** Overall survival. **c** Distant metastasis-free survival. **d** Loco-regional relapse-free survival

The 316 patients with stage III-IVb were divided into two subgroups: patients with a TTV ≤ 28 cm^3^ (*n* = 81) and patients with a TTV > 28 cm^3^ (*n* = 235). The 4-year estimated FFS, OS, DMFS, and LRRFS rates of the patients with a TTV ≤ 28 cm^3^ and TTV > 28 cm^3^ were 88.9 vs. 70.5% (*P* = 0.001), 96.2 vs. 72.7% (*P* < 0.001), 91.2 vs. 78.3% (*P* = 0.008), and 93.8 vs. 87.6% (*P* = 0.063; Fig. 4).

The following parameters were included in the Cox proportional hazards model: age (≤ 45 vs. > 45 years), sex (male vs. female), T category (T1–2 vs. T3–4), N category (N0–1 vs. N2–3), chemotherapy (yes vs. no), additional boost (yes vs. no) and TTV (≤ 28 vs. > 28 cm^3^). TTV was an independent prognostic factor for FFS, OS and DMFS in stage III-IVb NPC (all *P* < 0.05; Table 3).

**Table 3:** Multivariate analyses of prognostic factors in 316 patients with stage III-IVb

Endpoint	Variable	HR	95% CI	*P*-value
FFS	TTV	3.362	1.677–6.738	0.001
OS	Sex	1.735	0.906–3.321	0.096
	Additional boost	1.860	0.947–3.654	0.072
	Chemotherapy	2.384	1.109–5.126	0.026
	N stage^a^	1.551	0.994–2.421	0.053
	TTV	4.364	2.125–8.964	<0.001
DMFS	TTV	2.923	1.323–6.459	0.008
LRRFS	TTV	2.464	0.951–6.383	0.063

^a^According to the 7th edition of the American Joint Commission on Cancer staging system; *HR* hazard ratio, *CI* confidence interval, *FFS* failure free survival, *OS* overall survival, *DMFS* distant metastasis-free survival, *LRRFS* loco-regional relapse free survival, *TTV* total tumor volume, *RT* radiotherapy

### Prognostic validity of clinical stage combined with TTV vs. clinical stage alone for treatment failure

**Fig. 5:**
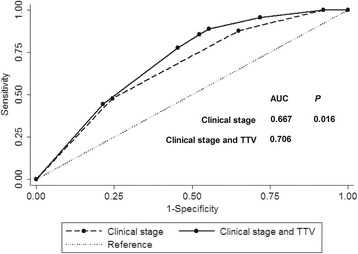
ROC curves for all patients stratified by clinical stage combined with TTV or clinical stage

ROC curves were used to compare the prognostic validity of clinical stage combined with TTV vs. clinical stage alone for treatment failure. The AUC for clinical stage combined with TTV was 0.706 compared to 0.667 for clinical stage alone (*P* = 0.016; Fig. 5). Therefore, the addition of TTV to clinical stage was superior to clinical stage alone for predicting treatment failure.

## Discussion

Tumor size is an important prognostic factor in cancer treatment and has been adopted in the staging systems for most carcinomas [19]. This NPC study demonstrated that patients with a TTV > 28 cm^3^ had significantly poorer survival outcomes compared to those with a TTV ≤ 28 cm^3^. Moreover, TTV was an independent prognostic factor in patients with NPC, and the addition of TTV to clinical stage was superior to clinical stage alone for predicting treatment failure.

### Distribution and optimal cut-off point for TTV

High TTV values were more frequent in patients with advanced clinical stage. However, the distribution of the TTV values varied widely within the same clinical stage, and overlapped between different clinical stages. Moreover, TTV, PTV and NTV exhibited large variations between different T and N categories [20, 24]. Our previous studies demonstrated that the distribution of the maximum primary tumor diameter (MPTD), another index of tumor size, exhibits a similar trend [25, 26]. Therefore, the current staging system for NPC has the disadvantage of assessing tumor size poorly.

Previous studies have divided patients into 2–4 groups on the basis of tumor volume using different methods [16, 27, 28]. Standard cutoff points should be adopted to achieve optimal sensitivity and specificity. For cancer patients at high risk of treatment failure, it is reasonable to maximize sensitivity over specificity. Therefore, we defined the ideal cut-off point based on a sensitivity estimate of over 80%. A cut-off point of 28 cm^3^ for the TTV was selected to assess treatment failure, and this cut-off value was validated in the test set.

### Prognostic value of the TTV in all patients with NPC

This study confirmed a large TTV was not only associated with poor FFS, DMFS and LRRFS, but also with poor OS in all patients with NPC. Chua et al. reported the 5-year FFS rates for patients with NPC treated by two-dimensional RT (2D–RT) with a TTV ≤ 20 cm^3^, > 20–40 cm^3^, > 40–60 cm^3^ and >60 cm^3^ were 89, 84, 76 and 55%, respectively (*P* < 0.001); and the corresponding 5-year DMFS rates were 84, 82, 73 and 61%, respectively (*P* < 0.001) [20]. Thus, it can be concluded that survival rates decrease with increasing tumor volume in patients with NPC treated by 2D–RT or IMRT.

Multivariate analyses showed TTV was an independent prognostic factor for FFS, OS, DMFS and LRRFS. In comparison, T category was an independent prognostic factor for OS, but not for FFS, DMFS or LRRFS. Furthermore, the only independent prognostic factor for LRRFS was TTV. Similar results were also observed in head and neck carcinomas: TTV was an independent variable, but T and N category were not independent prognostic variables unless the multivariate analyses did not include TTV [29]. In both this and the previous study, TTV appeared to be a more useful prognostic factor than the AJCC staging system. A large tumor volume may indicate a high potential for micro-metastasis, tumor hypoxia that promotes resistance to RT and chemotherapy, and an increased number of cancer clone cells to be killed [30, 31].

### Prognostic significance of the TTV in loco-regionally advanced NPC

This study also demonstrated that a large TTV was associated with poor FFS, OS and DMFS in stage III-IVb NPC. The TTV was also an independent prognostic factor for FFS, OS and DMFS in this group of patients. Our previous studies confirmed that MPTD is an independent prognostic variable in stage T3-T4 NPC [25, 26]. These findings indicate that although loco-regionally advanced disease is usually associated with a high risk of treatment failure, patients with advanced stage disease and a small tumor size may have a better prognosis.

Previous studies indicated the PTV was a significant prognostic factor for local control in NPC [20]. Sze et al. reported the risk of local control was estimated to decrease by 1% for every 1 cm^3^ increase in the PTV [16]. In this study, for patients with stage III-IVb NPC, TTV was only just statistically significant in LRRFS analysis. The main reasons for this observation may be as follows: first, with the development of IMRT, LRRFS has increased compared to patients treated with 2D–RT and 3-dimensional conformal radiation therapy [7, 8] and secondly, only 81 patients in the stage III-IVb group had a TTV ≤ 28 cm^3^. Therefore, this trend needs to be confirmed by analysis of a larger sample.

### Prognostic validity of adding TTV to clinical stage

The combination of TTV and clinical stage was superior to clinical stage alone for predicting treatment failure. Guo et al. reported prognostic assessment could be improved by combining the PTV with the current T classification criteria [21]. Our previous study also showed inclusion of the MPTD improved the prognostic value of the current T classification criteria [25]. Therefore, the current staging system for patients with NPC could be refined by incorporating tumor size.

In the clinic, the treatment strategy for NPC is mainly based on the name staging system, which lacks indexes related to tumor burden. TTV closely reflects tumor burden and is easily obtained from the IMRT planning system. As a large TTV was associated with a high incidence of treatment failure, patients with a large TTV may benefit from more aggressive treatment. For instance, adding induction chemotherapy, including cisplatin, fluorouracil, and docetaxel (TPF), to concurrent chemoradiotherapy could significantly improve FFS in locoregionally advanced NPC [32].

## Conclusions

This is the first evaluation of the prognostic value of the TTV in NPC, and reveals the TTV is an important prognostic factor for treatment outcomes in patients treated with IMRT. Incorporation of the TTV could help to refine the prognostic validity of the current staging system for NPC. Patients with a large TTV had a poor prognosis and may benefit from more aggressive treatment. However, this was a retrospective study of consecutive patients who received different chemotherapy regimens, which may have affected the treatment outcomes. Furthermore, this analysis was based on single-institution data, and needs to be confirmed via large-cohort multicenter studies.

## Abbreviations

AJCC: American Joint Commission on Cancer staging system
AUC: area under the curve, 2D, two-dimensional
CTV: clinical target volume
DMFS: distant metastasis-free survival
FFS: failure free survival
IMRT: intensity-modulated radiation therapy
LRRFS: loco-regional relapse free survival
MPTD: maximum primary tumor diameter
MRI: magnetic resonance imaging
NPC: nasopharyngeal carcinoma
NTV: nodal tumor volume
OS: overall survival
PTV: primary tumor volume
ROC: Receiver operating characteristic
RT: radiotherapy
TTV: total tumor volume
